# Cervical Regeneration Following Monopolar Electrosurgical Conization: A Prospective Evaluation of Volume, Length, and Transformation Zone Reformation

**DOI:** 10.3390/jcm14165918

**Published:** 2025-08-21

**Authors:** Şule Gül Aydın, Sevda Baş, Fatma Özmen, Şeyma Yaşar, Zeynel Abidin Taş, Ahmet Zeki Nessar, Sevtap Seyfettinoğlu, Mehmet Ali Narin

**Affiliations:** 1 Department of Obstetrics and Gynecology, Adana City Training and Research Hospital, Adana 01230, Turkey; 2 Department of Gynecologic Oncology, Adana City Training and Research Hospital, Adana 01230, Turkey; drsevdabas@gmail.com (S.B.); sevtaponcul@gmail.com (S.S.); mali_narin@yahoo.com (M.A.N.); 3 Department of Gynecological Oncology, Training and Research Hospital, Ordu University School of Medicine, Ordu 52000, Turkey; dr.fatmaozmenkaya@gmail.com; 4 Department of Biostatistics, İnönü University School of Medicine, Malatya 44280, Turkey; seyma.yasar@inonu.edu.tr; 5 Department of Pathology, Adana City Training and Research Hospital, Adana 01230, Turkey; zeynelabidin46@gmail.com; 6 Department of Perinatology, Osmaniye State Hospital, Osmaniye 80000, Turkey; zekinessar@gmail.com

**Keywords:** cervical intraepithelial neoplasia, cervical regeneration, monopolar electrosurgical conization (MESC), transformation zone, ultrasound

## Abstract

**Background:** The aim of this study was to evaluate the cervical regeneration process following monopolar electrosurgical conization (MESC), using a multimodal approach including ultrasonographic, cytologic, colposcopic, and histologic assessments, and to determine the relationship between the extent of excision and the capacity for cervical tissue regeneration. **Methods:** This prospective observational study included 28 patients who underwent MESC due to abnormal cervical cytology or biopsy-confirmed cervical intraepithelial neoplasia. Preoperative, postoperative one month and six month cervical measurements were obtained using two-dimensional transvaginal ultrasonography. Monthly colposcopic evaluations were conducted, cervical biopsies were taken at the third month, and a cytological assessment was performed at the sixth month. Cervical volume and length regeneration were calculated and analyzed in relation to the dimensions of the excised cone. Regeneration percentages and their correlations with excised tissue dimensions were evaluated using paired *t*-tests and Pearson correlation analysis. **Results:** Mean cervical volume and length regeneration rates at six months were 84.61% ± 5.64 and 86.36% ± 3.33, respectively. The transformation zone was histologically visible in 32.1% of patients at three months and cytologically in 75.9% at six months. An inverse correlation was observed between both cone volume and length and cervical regeneration (*p* < 0.005). Patients with larger preoperative cervical dimensions exhibited a higher regenerative capacity. Positive surgical margins were found in only one patient (3.4%), and no high-grade cytologic abnormalities were noted at follow-up. **Conclusions:** MESC may enable substantial cervical regeneration within six months. Larger excisions impair healing and delay transformation zone reformation, which may inform the optimal timing for initiating gynecological and colposcopic examinations, as well as for performing cervical interventions when indicated.

## 1. Introduction

The widespread implementation of cervical cancer screening programs has significantly reduced the incidence and mortality associated with cervical cancer [1]. According to the guidelines of the American Society for Colposcopy and Cervical Pathology (ASCCP), excisional treatments, rather than hysterectomy, are preferred for managing high-grade squamous intraepithelial lesions (HSIL), particularly in younger women [2,3].

Cervical excision methods include cold knife conization (CKC), laser conization, loop electrosurgical excision procedure (LEEP), and monopolar electrosurgical conization (MESC) [4]. The loop electrosurgical excision procedure (LEEP) is commonly associated with limitations such as reduced specimen volume, an increased risk of margin involvement, and tissue fragmentation. Consequently, in our institution, electrosurgical conization is performed using a monopolar cautery pen. This technique preserves tissue integrity and, owing to the larger excised tissue volume compared to the LEEP, reduces the probability of positive surgical margins [5].

During patient follow-up, the full visibility of the squamocolumnar junction represents a significant advantage, as it allows for adequate and reliable colposcopic surveillance [6]. This study aimed to investigate cervical tissue regeneration in terms of both volume and length following the MESC technique, and to determine the time required for re-epithelialization and transformation zone reformation. A combination of histological, colposcopic, and ultrasonographic methods was used to define the optimal timing for initiating gynecological and colposcopic examinations, as well as for performing cervical interventions when indicated.

## 2. Materials and Methods

This prospective observational study was conducted between September 2021 and December 2022 in the Gynecologic Oncology Clinic of Adana City Hospital. A total of 30 patients who had abnormal Pap smear results or were diagnosed with high-grade cervical intraepithelial neoplasia (CIN) based on colposcopic biopsy and underwent MESC, and who agreed to complete the 6-month follow-up protocol, were included in the study. Two patients were excluded from the analysis due to follow-up loss.

The inclusion criteria comprised patients aged between 18 and 65 years who were not pregnant at the time of admission, had abnormal cervical cytology or biopsy-confirmed cervical intraepithelial neoplasia (CIN 2-3) requiring excisional treatment, and had no history of prior cervical surgery, pelvic radiotherapy, or gynecologic malignancy. Exclusion criteria included a history of cervical surgery (such as repeat conization, ablation, cryotherapy, or subtotal hysterectomy) and any condition that altered the normal cervical anatomy (e.g., fibroids, cervical cancer, or masses).

Prior to the intervention, a detailed medical history was obtained from all patients. Information regarding age, parity, smoking status, presence of chronic diseases (such as diabetes mellitus), and use of medications (including steroids or immunosuppressive agents) was recorded.

Transvaginal ultrasonographic evaluation of the cervix was performed immediately prior to the MESC procedure, repeated at 1 month post-procedure and 6 months post-procedure, as described in various studies [7,8,9]. All ultrasonographic evaluations were performed by a single examiner to minimize bias. Sonographic examinations were performed by a 2D General Electric Logiq 500 Pro (GE Medical Systems, Milwaukee, WI, USA) scanner equipped with an E 721 endovaginal transducer (6 MHz). To obtain optimal imaging, patients were placed in the lithotomy position with an empty bladder. The transvaginal probe was advanced to the vaginal fornix without applying pressure to the cervix. During the transvaginal examination, the probe was positioned over the cervix to identify the internal os, external os, and cervical canal. In the sagittal plane, the distance between the internal and external os was measured as cervical length (L). The probe was then rotated 90° clockwise to measure the transverse diameter (D1) and anteroposterior diameter (D2) at the level of the largest axial plane of the cervix. Cervical volume was calculated using the cylindrical formula [7,10].

MESC was performed under general anesthesia with the patient in the lithotomy position. The procedure was carried out using a monopolar electrosurgical pen commonly utilized in open surgeries [11]. Prior to conization, hemostatic sutures using 1-0 absorbable material were placed at the 3 and 9 o’clock positions of the cervix to ligate the descending branches of the uterine arteries.

To minimize electrosurgical artifacts, the excision was performed in electrocision mode at 40 W. In case of bleeding, the device was switched to electrocoagulation mode for hemostasis. After adequate hemostasis was achieved, two connected vaginal tampons were inserted. The average duration of the procedure was 15 min under anesthesia. Following one day of inpatient monitoring, the tampons were removed, and patients were discharged. All procedures were performed by the same surgeon.

Conization specimens were sent for pathological evaluation. Volume measurements were performed in millimeters by the same pathologist using a ruler to measure length, width, and depth. Additionally, an Archimedes tube was used for volume calculation.

To assess cervical regeneration and the transformation zone, monthly colposcopic examinations were performed over the 6-month period, during which colposcopic photographs were obtained. At the third month, a cervical biopsy was performed under colposcopic guidance using Leech-Wilkinson forceps. Two types of biopsy sites were routinely selected: one at the interface between the unaffected squamous epithelium and the cauterized area, and the other at the center of the cauterized zone. Four-quadrant punch biopsies were taken from the junction between the healing area and the adjacent healthy tissue. Biopsies were obtained from the interface between the unaffected squamous epithelium and the cauterized area, covering all four quadrants (at 3–9 and 12–6 o’clock positions), as well as from the cauterized wound bed.

At the 6-month follow-up, cervical volume measurements were repeated via transvaginal ultrasonography using the same formula. Preoperative and postoperative 1 and 6 month cervical volumes and endocervical canal lengths were compared to calculate the percentage of regeneration. Volume regeneration percentage was calculated using the formula: [Conization volume − (Preoperative volume − Postoperative volume)] ÷ Preoperative volume × 100 and length regeneration percentage was calculated using the formula: [Conization length − (Preoperative length − Postoperative length)] ÷ Preoperative length × 100.

Data were summarized as mean ± standard deviation, median (min–max), and number (percentage). The Shapiro–Wilk test was used to assess the normality of the distribution. Depending on data characteristics, the Wilcoxon signed-rank test and the paired samples *t*-test were applied where appropriate. The direction and strength of the relationships between variables were assessed using the Pearson correlation coefficient. A *p*-value of <0.05 was considered statistically significant. All analyses were performed using IBM SPSS Statistics version 25.0.

## 3. Results

The mean age of the patients included in the study was 43.57 ± 9.14 years. Regarding cytological indications, the majority of patients were diagnosed with HSIL (70.4%), while LSIL and ASC-H were each observed in 14.8% of cases. In terms of histological indications, CIN2–3 was detected in 63.6% of patients, which was higher compared to CIN1 (36.4%). Pathological evaluation of the conization specimens revealed CIN1 in 46.4% and CIN2–3 in 39.3% of patients. Chronic cervicitis was identified in 14.3% of cases. Multiparity was highly prevalent: 96.4% of the patients were multiparous, and only 3.6% were nulliparous. With respect to HPV status, 66.7% of patients tested positive for high-risk HPV, while 33.3% had low-risk HPV results. Only 3.6% of the patients were diagnosed with diabetes mellitus, whereas 96.4% did not have diabetes. Regarding smoking status, 21.4% of patients were smokers, while 78.6% were non-smokers. Colposcopic biopsy results obtained to assess transformation zone (TZ) formation showed that TZ was not observed in 67.9% of patients and was present in 32.1%. According to cytological findings at the six-month follow-up, TZ was observed in 75.9% of patients. Following MESC, positive surgical margins were identified in only one patient, corresponding to 3.4% of the cohort (Table 1).

Prior to the MESC procedure, cervical volume and length measured by ultrasonography were 34.09 ± 3.35 cm^3^ and 33.77 ± 3.13 mm, respectively. The excised cone had a mean volume of 2.75 ± 1.08 cm^3^ and a length of 13.24 ± 3.10 mm. The means of volume and length regeneration were calculated as 84.61 ± 5.64% and 86.36 ± 3.33%, respectively (Table 2).

When cervical volume and length measurements at preoperative, one month and at six months were compared, statistically significant changes were observed in all parameters (*p* < 0.001). The means of cervical volumes at preoperative one month and at six months were 34.09 ± 3.35 cm^3^, 31.34 ± 4.42 cm^3^, and 33.61 ± 3.67 cm^3^, respectively. The volume deficit at one and six months was 2.75 ± 1.08 cm^3^ and 0.48 ± 0.32 cm^3^, respectively, and a statistically significant decrease was found (*p* < 0.001). The means of cervical lengths at preoperative, one month and at six months were 33.77 ± 3.13 mm, 20.53 ± 6.17 mm, and 31.86 ± 3.98 mm, respectively. Cervical length increased from 20.53 ± 6.17 mm at one month to 31.86 ± 3.98 mm at six months, and the length deficit showed a corresponding significant improvement (*p* < 0.001). Compared to preoperative values, the percentage changes in both volume and length also demonstrated significant differences (*p* < 0.001) (Table 3).

**Figure 1:**
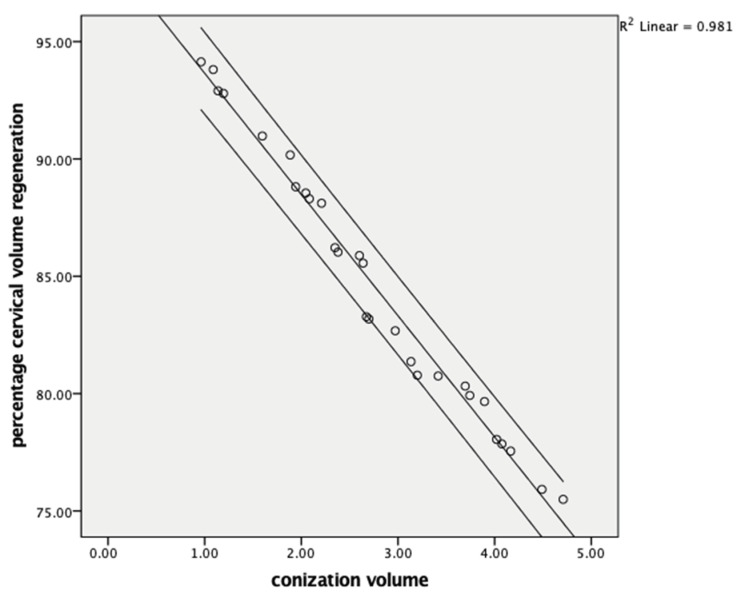
Comparison of the conization volume and cervical volume regeneration at six months.

**Figure 2:**
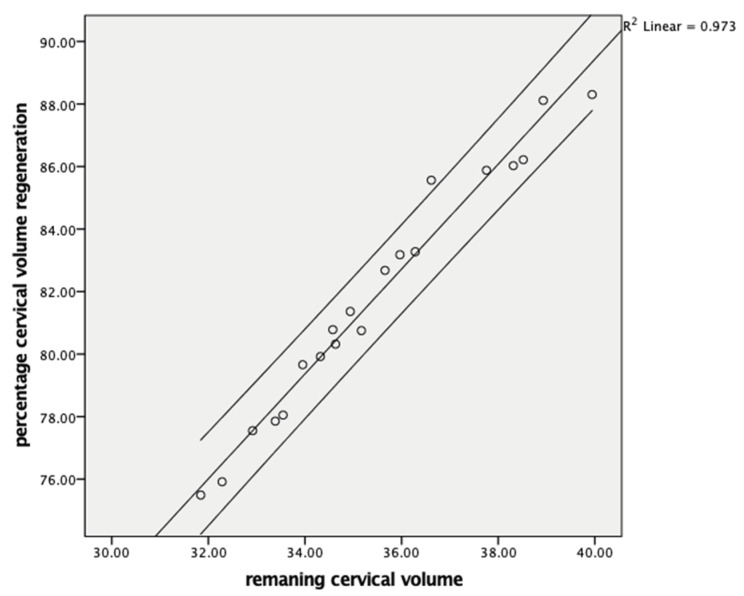
Comparison of the remaining cervical volume and cervical volume regeneration at six months.

Cervical volume regeneration showed a negative correlation with the resected cone volume (r = −0.990, *p* < 0.001) (Figure 1) and a positive correlation with the postoperative remaining cervical volume (r = 0.998, *p* < 0.001) (Figure 2). Cervical length regeneration showed a negative correlation with the resected cone length (r = −0.977, *p* < 0.001) and a positive correlation with the postoperative remaining cervical length (r = 0.473, *p* < 0.018) (Table 4).

## 4. Discussion

In the present study, we investigated cervical tissue regeneration after MESC both histologically and morphometrically, focusing on volume and length. Future studies comparing different conization techniques in this regard could offer important contributions to the literature.

This prospective observational study primarily aimed to investigate cervical tissue regeneration following monopolar electrosurgical conization (MESC), with a particular focus on volume and length recovery, as well as the timing of transformation zone (TZ) reformation.

A shortened cervical length has been associated with an increased risk of preterm delivery [7]. According to a multicenter case-control study, an excision depth exceeding 15 mm may be considered a risk factor for preterm delivery (relative risk: 2.04; 95% CI: 1.41–2.96) [12]. Although our study did not evaluate obstetric outcomes, its primary objective was to determine the timeframe required for cervical tissue healing following the MESC procedure. This knowledge is intended to guide the earliest safe timing for initiating gynecological and colposcopic examinations, as well as for planning further cervical interventions when necessary.

Our findings confirm that cervical regeneration is a consistent and measurable process, with mean regeneration rates of 84.61% for volume and 86.36% for length observed at six months postoperatively. These results are in line with those of previous studies. For instance, Founta et al. evaluated changes in cervical volume using magnetic resonance imaging (MRI) in 29 women, comparing pre-conization and six-month post-conization values. They reported that the mean cervical volume at six months corresponded to 97.8% of the baseline cervical volume, suggesting substantial regenerative capacity over time [13]. Papoutsis et al. also investigated the healing process of cervical defects following a LEEP for CIN using three-dimensional transvaginal ultrasonography between the pre-conization period and six months post-procedure in 73 women. They reported that the regeneration proportions of cervical volume and length after the LEEP were 81% and 78% of the initial measurements, respectively. Papoutsis et al. additionally examined the reparative process of cervical defects following the LEEP for cervical intraepithelial neoplasia (CIN) by employing three-dimensional transvaginal ultrasound imaging, conducted from the preoperative phase to six months post-procedure in a cohort of 73 women. They found that the restoration of cervical volume and length reached 81% and 78% of the original values, respectively [1]. Song et al. identified a statistically meaningful alteration in cervical volume and length when comparing baseline measurements to those at the 12 month follow-up. Their research highlights a gradual enhancement in cervical size both in volume and length persisting up to six months post-LEEP, as evidenced through a yearlong longitudinal evaluation [7]. Ciavattini et al. observed an inverse relationship between both the excised cone length and the proportion of cervical length removed and the degree of cervical length regeneration; however, they reported no such inverse association regarding cervical volume restoration [8]. Vincenzo Pinto et al. developed a measurement approach utilizing color doppler to pinpoint the location where the main branch of the uterine artery reaches the lateral border of the uterus. By the end of the six-month follow-up after conization, both cervical volume and length were found to regenerate comparably. The regeneration rates were recorded as 90.99% for volume and 91.07% for length, relative to the size of the excised cone [14].

In our study, the volume and length of the excised cone increased, and the regenerative potential of the cervix decreased. Strong and inverse correlations were identified between cone volume with volume regeneration and cone length with length regeneration. Cervical volume has been assessed in previous studies using three different modalities: three-dimensional ultrasound with VOCAL software, two-dimensional ultrasound, and magnetic resonance imaging (MRI) [9,13,15,16]. From a clinical standpoint, two-dimensional ultrasonography is more cost-effective and readily accessible compared to VOCAL-based 3D imaging and MRI, which facilitates its broader application in evaluating cervical volume [17]. In our study, cervical measurements were conducted using two-dimensional transvaginal ultrasonography. Despite employing a simpler technique, our findings were consistent with those obtained via three-dimensional ultrasonography and MRI, confirming that cervical volume regeneration is substantially achieved within six months following conization.

In many previous studies, the preferred method of conization has predominantly been the loop electrosurgical excision procedure (LEEP), with some also utilizing laser conization [8,15]. In contrast, our study employed the monopolar electrosurgical conization (MESC) technique using a monopolar electrode, and the observed rates of cervical regeneration were comparable to those reported with other excisional methods.

Ensuring complete visualization of the squamocolumnar junction during patient monitoring offers a notable benefit, as it facilitates thorough and dependable colposcopic assessments [6]. The purpose of this study was to evaluate cervical recovery following MESC by examining the epithelial regeneration process, specifically the renewal of squamous and endocervical layers and to determine the timeline for transformation zone reestablishment.

The transformation zone (TZ) was observed in 32.1% of patients at three months postoperatively. According to the control smear results obtained at the six-month follow-up, the visibility rate of the TZ increased to 75.9%, being observed in 22 patients. In 6 patients, the TZ was not visible on follow-up cytology; among these 6 patients, only 1 had a history of smoking, while no etiological factor could be identified to explain the delayed TZ regeneration in the remaining 5 cases. Sharp et al. utilized electron microscopy in their study investigating cervical epithelialization following laser conization and observed that epithelialization was completed by day 28. At the four-month follow-up, colposcopic examination and cytological sampling revealed that transformation zone formation and complete epithelialization had occurred in 29 out of 30 patients [18]. E. Bostofte et al. observed the timing of transformation zone formation in patients undergoing laser and cold knife conization using colposcopic evaluation. The visualization of the squamocolumnar junction was assessed via colposcopy at three and twelve weeks following conization. By the twelfth week, the squamocolumnar junction was entirely visible in 66% of patients who underwent laser conization, compared to 38% of those treated with cold knife conization [6]. Masamichi Kamasura et al. reported that following cryosurgical treatment of cervical erosion, flattening of the columnar epithelium became evident as early as the second day post-procedure. This was subsequently followed by reserve cell hyperplasia, which then progressed to squamous metaplasia within two to ten weeks after the intervention [19].

In their study observing cervical healing following laser conization, Jjnya Miyako et al. reported that endocervical epithelial repair of the squamocolumnar (S-C) junction tended to be delayed, and the newly formed S-C junction was located deeper within the endocervical canal compared to its original position. Based on their findings, they concluded that the regenerating epithelial cells likely originated from reserve cells located on the ectocervical side [20].

Columnar epithelial cells themselves do not undergo direct squamous metaplasia; they are supplanted by the proliferative activity of underlying subcolumnar cuboidal reserve cells. These endocervical glandular crypts may extend as deep as 5–6 mm beneath the cervical surface [21]. According to Anderson and Hartley, crypts can penetrate the stromal tissue up to 7.8 mm beneath the epithelial layer. Average depth of crypt involvement in the case of CIN was 1.24 mm, the extent of crypt invasion did not exceed 3.80 mm [22]. In the management of CIN, laser ablation does not need to exceed a depth of 4 to 5 mm. Controlled laser application provides sufficient vaporization depth while preserving the basal portion of the crypts, thereby promoting faster postoperative healing. Destruction to greater depths will lead to delays with healing because of the absence of the contribution from crypt epithelium [2,18]. Following excisional procedures such as LEEP, CKC or MESC, it may be postulated that a longer cone excision indicates more extensive involvement of the endocervical canal. This, in turn, could lead to the greater destruction of cervical reserve cells, thereby compromising the tissue’s capacity for adequate regeneration and repair [8]. In our study, the MESC technique was employed, with a mean excised cone depth of 13.4 ± 3.1 mm, corresponding to 40.31 ± 12.87% of the total cervical length. This extent of excision may have contributed to a delayed healing process and postponed formation of the transformation zone.

The LEEP is commonly associated with limitations such as an increased risk of margin involvement [23]. In the study by Jun Ding et al. comparing the LEEP and MESC techniques, the findings showed that the LEEP group had a significantly higher rate of positive endocervical margins (8.65% vs. 3.77%; *p* = 0.018) and thermal injury to the margins (10.38% vs. 4.90%; *p* = 0.016) [5]. In our study, surgical ectocervical margin positivity was observed in 1 out of 27 patients (3.4%).

## 5. Conclusions

This is the first study to describe the cervical regeneration process following monopolar electrosurgical conization (MESC) through a comprehensive approach combining histological, cytological, colposcopic, and ultrasonographic evaluations. Our findings demonstrate that cervical tissue exhibits a substantial capacity for regeneration within six months postoperatively, with mean recovery rates exceeding 84% for both volume and length. The extent of excised cone tissue was inversely correlated with regenerative outcomes, suggesting that more conservative excision may facilitate better healing. Furthermore, the transformation zone was reestablished in the majority of patients by six months, supporting the timing of follow-up examinations and further interventions. These results offer valuable insight into the healing dynamics after MESC and may aid in optimizing post-procedural surveillance strategies and clinical management of patients undergoing cervical excision.

## Figures and Tables

**Table 1:** Demographic and clinical characteristics of the study population.

Variable	n = 28
Age (years), Mean ± SD	43.57 ± 9.14
Cytology Indications, n (%)	
• LSIL	4 (14.8%)
• HSIL	19 (70.4%)
• ASC-H	4 (14.8%)
Histology Indications, n (%)	
• CIN1	4 (36.4%)
• CIN2–3	7 (63.6%)
Conization Pathology, n (%)	
• CIN1	13 (46.4%)
• CIN2–3	11 (39.3%)
• Chronic cervicitis	4 (14.3%)
Parity, n (%)	
• Nulliparous	1 (3.6%)
• Multiparous	27 (96.4%)
HPV Status, n (%)	
• Low-risk	1 (5.6%)
• High-risk	17 (94.4%)
Diabetes Mellitus, n (%)	
• No	27 (96.4%)
• Yes	1 (3.6%)
Smoking Status, n (%)	
• Non-smoker	22 (78.6%)
• Smoker	6 (21.4%)
3. month Biopsy, n (%)	
• TZ negative	19 (67.9%)
• TZ positive	9 (32.1%)
6. month Cytology-TZ Status, n (%)	
• TZ negative	6 (20.7%)
• TZ positive	22 (75.9%)
6. month Cytology Results, n (%)	
• NILM	25 (86.2%)
• ASC-US	1 (3.4%)
• LSIL	2 (6.9%)
Post-MESC Surgical Margin, n (%)	
• Negative	27 (93.1%)
• Positive	1 (3.4%)

Abbreviations: LSIL: Low-grade squamous intraepithelial lesion, HSIL: High-grade squamous intraepithelial lesion, ASC-H: Atypical Squamous Cells-cannot exclude HSIL, CIN: Cervical intraepithelial neoplasia, HPV: Human papilloma virus, TZ: Transformation zone, NILM: Negative for intraepithelial lesion or malignancy, ASC-US: Atypical squamous cells of undetermined significance, Post-MESC: Post-monopolar electrosurgical conization.

**Table 2:** Preoperative cervical and cone dimensions, and cervical volume and length regeneration measurements.

Variable	Mean ± SD
Preoperative cervical dimensions	
Cervical volume (cm^3^)	34.09 ± 3.35
Cervical length (mm)	33.77 ± 3.13
Excised cone dimensions	
Cone volume (cm^3^)	2.75 ± 1.08
Cone length (mm)	13.24 ± 3.10
Proportion Excised	
Proportion of volume excised (%)	8.45 ± 4.10
Proportion of length excised (%)	40.31 ± 12.87
Volume regeneration (%)	84.61 ± 5.64
Length regeneration (%)	86.36 ± 3.33

**Table 3:** Comparison of cervical volume and length measurements at preoperative one month and six months.

Variable	Preoperative	1. Month	6. Month	*p*-Value
Cervical volume (cm^3^)	34.09 ± 3.35	31.34 ± 4.42	33.61 ± 3.67	<0.001
% of baseline volume	100	91.55 ± 4.10	98.48 ± 1.15	<0.001
Deficit volume (cm^3^)	0	2.75 ± 1.08	0.48 ± 0.32	<0.001
Cervical length (mm)	33.77 ± 3.13	20.53 ± 6.17	31.86 ± 3.98	<0.001
% of baseline length	100	59.69 ± 12.87	94.09 ± 3.26	<0.001
Deficit length (mm)	0	13.24 ± 3.1	1.90 ± 0.89	<0.001

**Table 4:** Pearson correlation coefficients between cervical regeneration and related variables.

Variables	r (Volume Regeneration)	*p*-Value	r (Length Regeneration)	*p*-Value
Cone volume (cm^3^)	−0.990	<0.001		
Remaining cervical volume (post. MESC at 1. month)	0.998	<0.001		
Cone length (cm)			−0.977	<0.001
Remaining cervical length (post. MESC at 1. month)			0.473	0.018

## Data Availability

The datasets used and analyzed during the current study are available from the corresponding author on reasonable request.
